# Complementary Effects of Essential Oils and Organic Acids on Rumen Physiology as Alternatives to Antibiotic Feed Additives

**DOI:** 10.3390/ani15192910

**Published:** 2025-10-07

**Authors:** Rumbidzai Blessing Nhara, Joseph Jimu Baloyi

**Affiliations:** Department of Animal Science, Faculty of Science, Engineering and Agriculture, University of Venda, Private Bag X5050, Thohoyandou 0950, South Africa; joseph.baloyi@univen.ac.za

**Keywords:** alternative feed additives, essential oils, organic acids, complementary effect, rumen physiology

## Abstract

The investigation into the complementary roles of essential oils (EOs) and organic acids in enhancing rumen physiology is increasingly gaining recognition within the field of animal nutrition. Essential oils are known for their antimicrobial effects, which can specifically target certain microbial populations in the rumen, thereby impacting fermentation processes, methane output, and nutrient digestion. In addition, the integration of organic acids plays a crucial role in stabilizing rumen pH and steering the metabolic activities of bacterial populations toward propionate production, a process essential for energy metabolism in ruminants. The concurrent use of essential oils and organic acids may yield synergistic benefits that could further optimize ruminal fermentation efficiency, enhance feed conversion rates, and lower methane emissions. This systematic review used the PRISMA (Preferred Reporting Items for Systematic Reviews and Meta-Analyses) guidelines. The literature search was meticulously designed to encompass parameters related to ruminant species, feed additives, essential oils, organic acids, synergistic effects, and rumen physiology. The efficacy of both organic acids and essential oils is highly dependent on their concentration and the specific combinations utilized. When certain essential oils are used in conjunction with selected organic acids, they may mitigate any potential negative effects on fermentation, thereby fostering a more favorable environment for the proliferation of beneficial microbial communities. Understanding the relationship between essential oils and organic acids is essential for the formulation of diets that enhance rumen health while concurrently reducing environmental pressures through diminished methane emissions. Future research should prioritize long-term in vivo investigations to gain more comprehensive insights into the interactions among these dietary components and identify the optimal combinations for ruminant feeding strategies.

## Simple Summary

In animal nutrition, there is growing interest in the complementary roles that organic acids and essential oils (EOs) play in regulating rumen physiology. EOs are known to have antibacterial qualities that can selectively inhibit rumen microbial groups, affecting methane generation, nutrient digestion, and fermentation dynamics. The use of organic acids aids in rumen pH maintenance and promotes the metabolic pathways of bacteria that lead to the synthesis of propionate, which is essential for ruminant energy metabolism. A synergistic impact with essential oils may further increase the efficiency of ruminal fermentation, increase feed conversion, and lower methane emissions. The efficacy of organic acids and essential oils is highly dependent on their concentration and the precise combinations used. The use of essential oils in conjunction with certain organic acids may counterbalance any negative effects on fermentation by creating a more favorable environment for beneficial microbes to thrive. Understanding the interplay between essential oils and organic acids is critical for optimizing dietary formulations that promote better rumen health while minimizing environmental impacts through reduced methane emissions. Future studies should focus on long-term in vivo investigations to gain a better understanding of the dynamics of these dietary additives and their best combinations for ruminants.

## 1. Introduction

The increase in public and scientific concerns about the use of antibiotics as feed additives in animal production has led to a real demand for alternative feed additives in animal production [1]. Alternative feed additives which include organic acids involved in metabolic pathways and plant extracts like essential oils can offer benefits provided by antibiotic feed additives and reduce the risk associated with antimicrobial resistance (AMR) [2]. The manipulation of rumen physiology through dietary additives, such as essential oils, organic acids and their blends, has gained considerable attention in enhancing ruminant health and productivity [3]. Feed additives, when included in ruminant diets, have specific targets, which include reducing methane production in favor of propionate to improve the energy balance in animals, reducing feed protein degradation to increase the bioavailability of amino acids in the small intestines, reducing the degradation rate of rapidly fermentable carbohydrates and control lactic acid concentrations and lastly, improving fiber digestion [4]. Essential oils (EOs) are recognized for their antimicrobial properties, which can alter the microbial ecology of the rumen, impacting fermentation processes, methane production, and overall health of ruminants [2]. Organic acid feed additives have an influential factor in enhancing rumen physiology and fermentation processes in ruminants by modulating the microbial community and metabolites produced [5]. Recent studies indicate that essential oils can effectively reduce methane emissions in the rumen by inhibiting methanogenic archaea and altering the fermentation pathways toward more propionate and butyrate production [6]. Abdillah et al. [7] demonstrated that nutmeg essential oil significantly decreased methane production, suggesting that its bioactive components can inhibit hyper ammonia-producing bacteria, thereby reshaping the microbial population in the rumen environment [8]. Additionally, rosemary essential oil has been noted to reduce ammonia concentrations by acting on specific bacterial populations, influencing volatile fatty acid (VFA) profiles that are critical for ruminant metabolism [9]. Specific doses of EOs are found to enhance the growth of beneficial bacteria such as Fusobacterium succinogenes (*F. succinogenes*, critical for fiber digestion, while hindering more harmful bacteria like *Selenomonas* [10]. In addition to essential oils, organic acids such as malate and fumarate have been shown to stabilize rumen pH, thus supporting a favorable environment for microbial activity and propionate production, which is vital for enhancing energy efficiency in ruminants [11]. While the application of essential oils and organic acids shows great promise, the variability in responses based on dose, species, and specific microbial communities necessitates further research to maximize their efficacy. This variability is underscored by differences in studies often attributable to different experimental designs and the chemical composition of the essential oils or organic acids used [12]. A complementary approach of combining essential oils and organic acids presents a viable strategy for enhancing rumen physiology, reducing methane emissions, and improving the overall health and performance of ruminant animals [13,14,15]. The focus of this systematic review is to provide a comprehensive account of essential oils and organic acid blends as alternative feed additives in ruminant animals and how they will impact rumen physiological parameters.

## 2. Materials and Methods

### 2.1. Eligibility Criteria

The eligibility criteria for the article focused on studies involving essential oils and organic acid feed additive in ruminants, specifically targeting the use of one or more additives in ruminant diets. Eligible studies must provide quantitative data on at least one rumen physiology parameter, such as rumen pH, rumen microbiome, methane production, volatile fatty acids or rumen development. Only original experimental studies and reviews were included, excluding abstracts, and non-English articles. Additionally, the timeframe for publication was set between 2010 and 2025.

### 2.2. Search Strategy

A systematic literature review was conducted using Web of Science, Google Scholar, Scopus, and PubMed to gather peer-reviewed papers from 1990 to 2025. The methods employed in this systematic review were based on the PRISMA (Preferred Reporting Items for Systematic Reviews and Meta-Analyses) statement [16]. The search criteria focused on ruminant animals, antibiotic feed additives, alternative feed additives, essential oils, organic acids, complementary effects and rumen physiology. The data was analyzed to determine if essential oils and organic acids blends can be used as alternative to antibiotic feed additives in ruminant diets and to understand how their complementary effect affects rumen physiology. Data was analyzed, synthesized, and presented based on the key questions raised in the development of a review.

### 2.3. Article Selection Criteria

The standards established for identifying eligibility and inclusion in this review were studies centered on essential oil and organic acid blends utilized in ruminant diets, covering a period of fifteen years, from 2010 to 2025, and published in English. Table 1 below further explains the selection criteria using the PICO method.

### 2.4. Data Extraction

Study information was gathered and carefully evaluated to ensure agreement on all essential aspects. To preserve the accuracy and quality of the data, every article was subjected to an in-depth examination, and the relevant details were meticulously recorded. The data that was extracted and recorded from all the selected articles included the type of additive used, its concentration/inclusion level, and the reported findings.

## 3. Characteristics and Properties of Essential Oils and Organic Acids

Essential oils and organic acids are recognized for their diverse characteristics and properties, making them valuable in various applications, particularly in medicine, food preservation, and agriculture [16]. Essential oils are concentrated plant extracts primarily composed of terpenes and their oxygenated derivatives, termed oxyterpenes, along with minor quantities of other organic compounds such as aliphatic alkanes, alcohols, aldehydes, ketones, and organic acids [17,18]. These unique properties and their interaction with animal biochemical and hormonal pathways extend to various physiological effects, including antimicrobial actions, where essential oils can inhibit the growth of pathogens while preserving beneficial bacteria within the intestinal microbiome [19,20]. Additionally, essential oils exhibit significant antioxidant activity, as evidenced by their ability to scavenge free radicals and suppress reactive oxygen species (ROS) [20]. This property is crucial to promoting health in animals by mitigating oxidative stress in biological systems [21].

Organic acids are commonly used in food technology and animal feed due to their versatile functionalities [22]. In animal husbandry, organic acids have been shown to enhance growth performance in poultry by modulating gut microbiota, improving feed conversion rates, and stimulating digestive enzyme activity [23,24]. Furthermore, the combination of organic acids can produce synergistic antimicrobial effects, enhancing their overall efficacy compared to singular use [25]. This synergy arises from the varying spectra of activity and mechanisms of action across different acids, making them preferable in complex dietary formulations in livestock [26].

**Figure 1:**
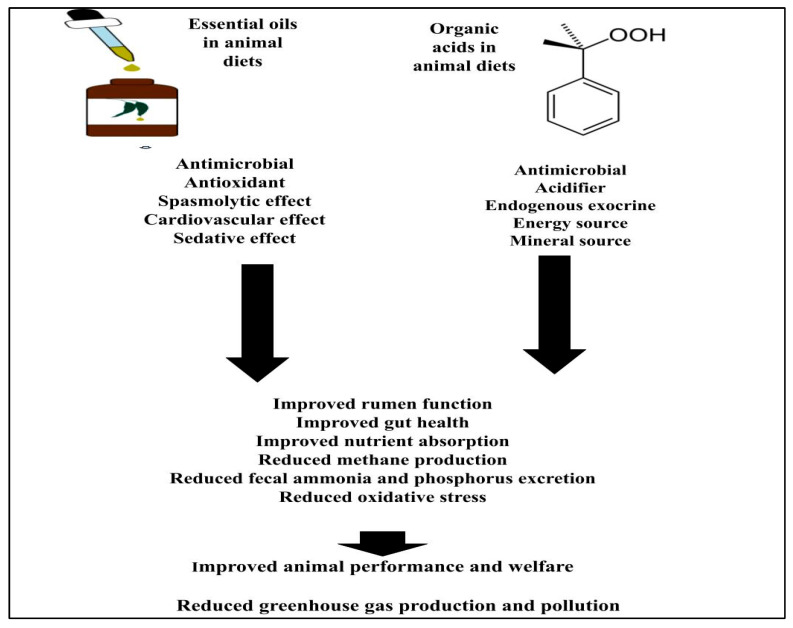
Summary of properties of essential oils and organic acids and the potential effect on animal performance and environment.

The integration of essential oils and organic acids offers a complementary approach in various applications and the combined antimicrobial properties of both can enhance the suppression of bacterial growth in the GIT [27]. The synergistic effects of essential oils and organic acids can significantly improve gut health and animal performance by collectively enhancing the overall microbial environment [28]. Essential oils and organic acids present a range of beneficial characteristics and properties as shown in Figure 1, which not only serve functional roles in their respective applications but also hint at their collaborative potential in enhancing animal performance, health and safety standards [28].

## 4. Impact of Antibiotics and Ionophores on Rumen Physiology

Rumen physiology is significantly influenced by the dietary inclusion of antibiotics and ionophores, which play an essential role in modulating microbial populations and fermentation processes within the rumen [29]. Ionophores primarily act by selectively inhibiting Gram-positive bacteria, including hyper-ammonia-producing bacteria that negatively affect protein utilization in ruminants. Studies have indicated that the addition of monensin can diminish ammonia formation by targeting these Gram-positive bacteria, effectively altering the microbial balance in the rumen [29,30]. Ionophores inhibit gram positive bacteria and promote propionate production, a critical volatile fatty acid (VFA) serving as a key energy source that supports improved hepatic gluconeogenesis, thereby enhancing overall energy efficiency in livestock [12]. Ionophores like monensin can modify rumen fermentation patterns, resulting in increased propionate concentration and reduced acetate and butyrate levels during fermentation. These changes not only improve production efficiency but also lead to enhanced average daily gain, reduced feed intake, and better feed conversion rates, which are vital metrics for livestock productivity [31]. They have been found to significantly reduce methane emissions, which is advantageous from an environmental perspective [32,33,34]. The increasing interest in natural feed additives is also linked to concerns regarding antibiotic resistance, a notable issue associated with the prolonged use of ionophores and traditional antibiotics [35,36]. However, the effects of ionophores and antibiotics in ruminant feed can vary based on numerous factors, including the type of animal, diet composition, and physiological state [37].

## 5. Potential Effect of Essential Oils and Organic Acids in Animal Nutrition

### 5.1. Rumen Physiology

Rumen physiology is a complex interplay of microbial fermentation, host interactions, and metabolic processes, which are crucial for nutrient absorption and energy homeostasis [38,39]. The rumen serves as the first chamber in the ruminant stomach, primarily responsible for the fermentation of ingested food aided by a diverse microbial community [40,41]. This fermentation process results in the production of volatile fatty acids (VFAs), which are vital energy sources for the host and facilitate various physiological functions, including growth and maintenance of the rumen epithelium. Dietary composition is one of the key aspects that influence rumen physiology, particularly the types of carbohydrates and fibers present [40,42]. High-fiber diets encourage the growth of fiber-degrading bacteria, enhancing cellulose breakdown and VFA production, thereby promoting a healthy rumen environment. Conversely, diets rich in non-fiber carbohydrates, such as starch, can lead to subacute ruminal acidosis (SARA), which is detrimental as it alters the rumen pH and disrupts microbial homeostasis [3,41]. A low pH generally favors the growth of lactate-producing bacteria, which can precipitate acidosis, damage the rumen lining, and ultimately impair nutrient absorption. The role of dietary fermentative processes is also critical in developing the rumen infrastructure, particularly in young ruminants [42]. The introduction of solid feed stimulates growth in the rumen papillae, improving surface area for nutrient absorption and enhancing the microbial ecosystem [43]. This transformation is supported by the microbial metagenome, which co-evolves with the host transcriptome, suggesting a profound relationship between diet, microbial populations, and ruminant physiology.

Rumen health is closely associated with the integrity of the rumen epithelial barrier, which can be compromised by inappropriate dietary choices [44]. High-sulfur diets have been shown to induce keratinization and tissue damage, thereby increasing the permeability of the rumen wall to pathogens. This can lead to systemic issues as toxic substances may breach the epithelial barrier, complicating the physiological state of the animal. Short-chain fatty acids (SCFAs) in diets are also important for nutrient absorption and energy supply for the epithelial cells of the rumen [45]. These metabolic byproducts of fermentation are critical for the host’s energy requirements and modulate significant metabolic and physiological pathways in ruminants, shaping the overall rumen function [39]. The physiology of the rumen is multifaceted, involving the intricate balance between diet, microbial activity, and host health.

### 5.2. Complementary Effect of Essential Oils and Organic Acids on Rumen Development

The development of rumen papillae and the subsequent nutrient absorption capacity are significantly influenced by dietary factors, including essential oils (EOs) and organic acids [46]. These changes are critical in improving rumen health and maximizing nutrient absorption in ruminants. Rumen papillae, which increases the surface area available for nutrient absorption, undergoes morphological changes in response to dietary composition, particularly regarding the presence and concentration of VFAs such as propionate and butyrate [47]. Studies indicate that VFAs constitute essential energy sources, meeting approximately 70–80% of the energy requirements for the epithelial development in ruminants. The presence of these acids significantly promotes the structural development and functional capacity of the rumen epithelium, enhancing both its absorptive surface and nutrient absorption efficiency [48,49,50,51]. Essential oils, recognized for their antimicrobial properties, can modulate the ruminal microbial population, subsequently influencing VFA profiles and rumen fermentation patterns [2]. Certain EOs have been demonstrated to enhance the production of beneficial fermentation end-products while suppressing harmful bacteria, promoting a healthier rumen environment [4,50]. This interaction not only boosts the efficiency of rumen fermentation but also aids in the structural integrity of the rumen epithelium by creating a conducive environment for VFA production [50]. Thus, essential oils can indirectly facilitate the development of rumen papillae by optimizing the microbial fermentation process that generates these critical acids. Essential oils and organic acids in diets profoundly affect rumen papillae development and absorption efficacy by shaping microbial communities and enhancing VFA production [2]. The structural adaptations of the rumen epithelium, driven by these dietary factors, are crucial for improving nutrient uptake and overall animal performance during the transition to solid feeds.

### 5.3. Complementary Effect of Essential Oils and Organic Acids on Rumen Environment

The complementary effects of essential oils and organic acids on the rumen environment are an emerging area of study with implications for ruminant health and productivity. Essential oils, known for their antimicrobial properties, and organic acids, which can enhance fermentation efficiency, both play significant roles in modifying rumen fermentation processes and can synergistically improve animal performance [2]. Organic acids, such as fumaric acid, have been increasingly utilized in ruminant nutrition, primarily as alternatives to antibiotics [5]. Their ability to reduce methane emissions and improve the efficiency of feed utilization is documented. Studies indicate that organic acids can prevent energy waste in feeds by altering fermentation patterns in the rumen, leading to enhanced digestibility and lower ruminal methane production [51]. Dicarboxylic acids like malate and fumarate are particularly notable for their ability to attenuate pH drops during high grain feeding, as evidenced by [52]. These acids facilitate lactic acid uptake and enhance the succinate-propionate pathway in rumen bacteria, resulting in improved fermentation efficiency and propionate production, which is vital for ruminant metabolism. However, there is a significant gap in understanding the synergistic effects of different organic acids when used with other feed additives, such as essential oil [13,53]. Essential oils contain bioactive compounds that can modulate the microbial population in the rumen, thereby influencing fermentation outcomes. The incorporation of these oils into the diet has been shown to enhance fiber degradation and increase the production of beneficial volatile fatty acids (VFAs) [54]. Specific essential oils can select for beneficial rumen microbes like *Butyrivibrio* and *Ruminococcus,* which support fiber digestion and VFA production [55,56]. The interplay between essential oils and organic acids may therefore create an optimal rumen environment that promotes efficient fermentation while mitigating undesirable microbes [13]. Research has identified that the balance between different VFAs can be manipulated by the types of feed additives used. For example, certain organic acids can increase the concentration of butyric acid while decreasing propionic acid levels, thereby affecting the overall energy balance in ruminants [5]. Moreover, maintaining optimal rumen pH is critical for maximizing digestion and preventing acidosis, which is a risk that increases with high-starch diets typical in concentrate feeding [57]. Essential oils complemented with organic acids may help stabilize rumen pH by enhancing microbial activity and buffering capacity [13]. Despite these benefits, challenges remain regarding the precise mechanisms through which these additives interact in the rumen environment. Understanding their individual and combined effects will require more comprehensive studies focused on microbial dynamics and fermentation outcomes [58]. Additionally, the potential for enhancing animal health through the modulation of rumen microbiota using these natural compounds offers a promising avenue for developing sustainable ruminant feeding strategies [59,60]. The integration of essential oils and organic acids may offer significant improvements in rumen fermentation efficiency and animal productivity, though further research is necessary to elucidate the complex interactions and optimal inclusion rates for these additives.

### 5.4. Complementary Effect of Essential Oils and Organic Acids on Microbial Ecology

The interplay between essential oils (EOs) and organic acids significantly influences the microbial ecology of the rumen, a crucial digestive system compartment in ruminants. Essential oils possess unique bioactive compounds that modulate the microbial populations in the rumen, predominantly through their antimicrobial properties. Research indicates that essential oils affect the functional dynamics of the rumen microbiome, often mimicking the action of ionophores by selectively inhibiting methanogenic microbes while promoting the growth of fiber-degrading bacteria [61]. The essential oil from rosemary leaves has shown differential effects compared to its solid form, primarily due to variations in chemical composition that influence antimicrobial activity [14]. This suggests that the mode of supplementation—whether as an oil or dried herb—can significantly impact the microbial ecology. In vitro, studies have demonstrated that garlic oil effectively reduces enteric methane emissions from buffaloes, showcasing the potential of specific essential oils to inhibit methanogenic archaea in the rumen [62]. Notably, certain plant oils such as coconut oil have been reported to possess properties that may suppress methanogenic populations, thus contributing to lower methane production [63]. Organic acids, particularly those derived from fermentation processes, also exhibit properties that influence rumen microbial dynamics. Studies suggest that dietary acids, especially medium-chain fatty acids, can suppress bacterial populations associated with cellulolytic activity and methanogenesis [64]. The manipulation of pH through the inclusion of these acids in the diet can create an environment conducive to beneficial microbial growth while inhibiting fewer desirable species [65]. The integration of essential oils with organic acids holds promise for optimizing rumen fermentation, as evidenced by various studies illustrating the ability of specific EOs to enhance digestibility while simultaneously suppressing methane production [66]. Improving feed efficiency and reduction in greenhouse gases can benefit both animal production and environmental sustainability [67]. Moreover, essential oils from plants like oregano have been shown to promote beneficial microbial populations (*R. flavefaciens*, *R. albus* and *F. succinogenes*), while reducing overall methane emissions, making them effective natural alternatives to chemical additives [68]. However, it is crucial to consider that higher concentrations of certain EOs can lead to detrimental effects on ruminal fermentation, necessitating careful dose regulation [69]. The variability in effects based on dietary composition further emphasizes the need for tailored supplementation strategies [70]. The complementary effects of essential oils and organic acids on rumen microbial ecology can significantly enhance rumen fermentation efficiency while mitigating methane production.

### 5.5. Complementary Effect of Essential Oils and Organic Acids on Rumen Fermentation Parameters

The interaction of essential oils and organic acids in altering rumen fermentation parameters has garnered significant attention in recent studies, reflecting their potential to enhance ruminant productivity and mitigate undesirable outcomes such as methane emissions. Essential oils, characterized by their volatile nature and bioactive compounds, can significantly modulate rumen microbial communities and fermentation outcomes. Research has shown that essential oils, derived from eucalyptus and oregano, exhibit antimicrobial properties that can reduce methanogenesis through the restructuring of microbial populations within the rumen [66,68]. The addition of essential oils often results in a shift in volatile fatty acid (VFA) profiles, promoting the production of propionate over acetate, consequently improving feed efficiency and reducing methane emissions [71]. High concentrations of specific essential oils may, however, negatively impact total VFA production, suggesting a threshold beyond which beneficial effects diminish [71].

Moreover, the inclusion of organic acids in ruminant diets has been documented to influence fermentation parameters by enhancing energy availability and stabilizing rumen pH [72]. Organic acids like acetic, propionic, and butyric acids play critical roles in energy metabolism, where their supplementation positively influences microbial dynamics leading to improved nutrient digestion [72]. Furthermore, the combination of essential oils with organic acids can synergistically enhance rumen fermentation efficiency while mitigating risks associated with suboptimal pH conditions, such as acidosis [15]. This interplay between organic acids and essential oils may offer a practical approach to optimize rumen function under varying dietary conditions.

The fatty acid profile of dietary oils also merits consideration in understanding their fermentative effects. Research indicates that medium-chain fatty acids from oils like palm and coconut can suppress methanogenic activity while maintaining the overall performance of rumen microbial populations [73]. The adaptability of microbial flora to these dietary changes has been noted, underscoring the resilience of the rumen ecosystem in accommodating different feeding strategies without compromising fermentation efficiency [74].

### 5.6. Complementary Effect of Essential Oils and Organic Acids on Absorption and Nutrient Uptake

The rumen structure and its absorption capacity are influenced by several interrelated factors, including dietary components, genetic variations, microbial activity, and environmental conditions. Understanding these factors is essential for optimizing ruminant nutrition and improving overall livestock productivity. One critical determinant of rumen health and function is the diet composition, particularly its fiber and carbohydrate content. High-grain diets tend to reduce ruminal pH due to increased fermentation activity, resulting in the production of short-chain fatty acids (SCFAs) which can lead to conditions like subacute ruminal acidosis (SARA) when their production outpaces absorption capacity [75,76]. The rumen epithelium’s ability to absorb SCFAs is contingent upon maintaining an optimal pH range, which is generally around 6.0 to 7.0; deviations below this range can impair nutrient digestion and absorption by affecting epithelial cell integrity and function [77]. Enhanced expression of transporters, such as the monocarboxylate transporter (MCT1) and sodium–hydrogen exchanger isoform 3 (NHE3), is observed in response to low-pH conditions to facilitate SCFA absorption despite the acidic environment [78]. The interaction between host genetics and diet can create differences in rumen microbial communities, thereby affecting fermentation patterns and the extent of VFA absorption [75]. The health of the rumen epithelium is crucial as it is responsible for absorbing nutrients, including SCFAs, which are vital for the energy supply of ruminants [79]. Enhanced papillae length and thickness resulting from appropriate diet and supplementation positively correlate with improved absorption rates and overall nutrient efficiency [80]. Additionally, certain essential oils, such as those from eucalyptus and nutmeg, have displayed potential in altering enzymatic activity and metabolite profiles in the rumen, indicating a broader impact on nutritional uptake and health [71]. Essential oils can improve protein metabolism by reducing ammonia levels in the rumen and enhancing nitrogen utilization efficiency, as evidenced by studies examining basil oil effects on buffalo calves [81]. The rumen’s structural integrity and efficiency in nutrient absorption are products of dietary choices, genetic factors, microbial health, and environmental conditions. Each of these elements plays a role in maintaining the delicate balance necessary for effective rumination and overall cattle health. Well-designed supplementation strategy utilizing both essential oils and organic acids could enhance nutrient assimilation efficiency in ruminants, ultimately translating to improved growth performance and production metrics in livestock.

### 5.7. Complementary Effect of Essential Oils and Organic Acids on Rumen Degradation and Nutrient Digestion in the Abomasum

The interaction between organic acids, essential oils, and their impact on rumen degradation and nutrient digestion in the abomasum is an area of increasing interest in animal nutrition. Organic acids and essential oils modulate the rumen environment, thereby affecting fermentation processes, nutrient availability, and ultimately digestibility in the abomasum.

Organic acids, such as those derived from fruits or microbial fermentation, influence rumen fermentation by altering pH and improving the overall microbial profile [5]. The addition of medium-chain fatty acids as organic acid blends has been linked to improved nutrient digestibility and absorption in various studies. Dietary supplementation with organic acids led to significant benefits in nutrient digestibility, particularly in the ileum and total tract digestibility in growing pigs, due to the acidifying effect that enhances overall digestion processes [82]. This supports the notion that organic acids can enhance the release of nutrients trapped in complexes with tannins in the rumen, allowing for better absorption in the more acidic abomasum environment [83].

Furthermore, fermentation in the rumen produces volatile fatty acids (VFAs), which are crucial for ruminants’ metabolism and influence growth performance [84]. The subsequent passage of these volatile fatty acids into the abomasum can significantly impact energy utilization. The ruminal pH optimally supports the growth of beneficial bacteria, while the lower pH in the abomasum helps dissolve nutrient complexes, enhancing protein digestibility through the breakdown of protective tannin-nutrient complexes found in certain feedstuffs like soybean meal and legumes [83].

**Figure 2:**
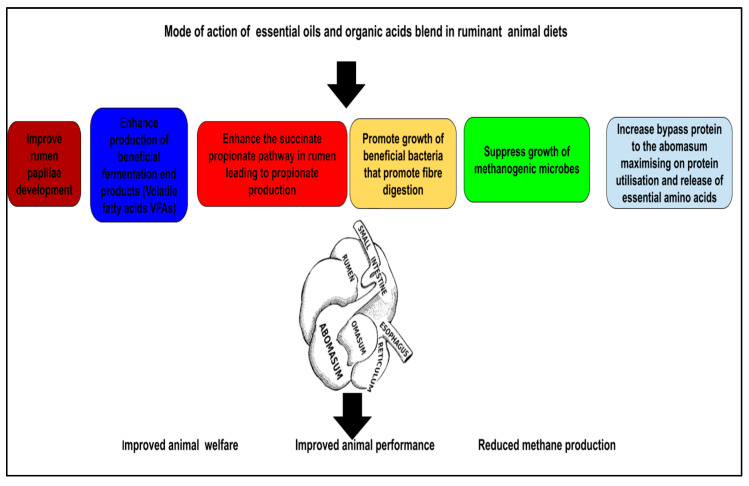
Mode of action of essential oils and organic acid blend on rumen physiology and the gastrointestinal tract.

Essential oils, which contain various bioactive compounds, modify rumen fermentation profiles. They have been shown to promote the growth of beneficial bacteria while inhibiting pathogens, enhancing nutrient utilization efficiency [82]. The strategic use of specific essential oils in ruminant diets can also improve the nutrient flow from the rumen to the abomasum, where digestive processes are further enhanced. Essential oils may stimulate gastric secretions that promote the emulsion of fats and proteins, enabling better assimilation and reducing nutrient losses to the lower gut [85]. Moreover, dietary components such as protein sources are underscored by research indicating that protected proteins can bypass the rumen to the abomasum effectively [86]. This is essential for maximizing protein utilization in ruminants, and organic acids can facilitate the release of amino acids from such diets [87,88]. Studies indicate that, through environmental manipulation in the rumen, organic acids can increase the apparent availability of bypass proteins by altering microbial fermentation dynamics and nutrient interactions [89,90]. Combined effects of organic acids and essential oils through enhanced fermentation and nutrient dissociation in the gastrointestinal tract, present promising avenues for improving feed efficacy and animal performance in ruminants. Figure 2 summarize of mode of action of essential oils and organic acids feed additives in ruminant animal diets. 

## 6. Future Perspective and Conclusions

The regulation of rumen physiology through dietary supplementation with essential oils (EOs) and organic acids presents a promising avenue for enhancing livestock productivity and sustainability. EOs have been shown to exert antimicrobial effects on rumen microorganisms, thereby altering fermentation patterns and potentially reducing methane emissions [91]. Organic acids can modify the rumen environment and microbial communities [5]. Moreover, dietary oils, particularly those rich in medium-chain fatty acids, may provide additional benefits by suppressing methanogenesis. It is also important to recognize that high levels of unsaturated fatty acids may disturb rumen fermentation, especially when not balanced with adequate fiber availability, which is crucial for maintaining microbial activity [92]. In addition to fermentation dynamics, the implications of these dietary strategies for animal health must be considered. The strategic integration of both essential oils and organic acids in diets could therefore create a synergistic effect, optimizing fermentation, enhancing nutrient absorption, and improving overall rumen health. The exploration of complementary effects from essential oils and organic acids on rumen physiology underscores a critical intersection between nutrition and microbiology in ruminant production systems. Future research should focus on the long-term effects of these dietary interventions on animal welfare, productivity, and environmental sustainability, as well as the mechanistic pathways through which these compounds exert their beneficial effects on rumen microbial communities.

## Figures and Tables

**Table 1:** PICO explanation of the article selection method.

PICO Element	Inclusion Criteria	Exclusion Criteria
Population	Essential oil and organic acid feed additives blend	Other feed additives
Intervention	Additives in ruminant animal diets	Monogastric animals
Comparator	Studies with control groups	No control groups
Outcomes	Quantitative data, reviews on rumen physiology parameters (rumen pH, rumen microbes, methane production)	Qualitative studies, reviews, and abstracts without complete data integrity
Study design	Original experimental studies, systematic reviews, meta-analysis	Case reports, editorials, conference abstracts without full methods
Study period	Studies from 2010 to 2025	Studies before 2010

## Data Availability

Not applicable.
